# Identification of Widespread Antibiotic Exposure in Patients With Cholera Correlates With Clinically Relevant Microbiota Changes

**DOI:** 10.1093/infdis/jiz299

**Published:** 2019-06-13

**Authors:** Ludmila Alexandrova, Farhana Haque, Patricia Rodriguez, Ashton C Marrazzo, Jessica A Grembi, Vasavi Ramachandran, Andrew J Hryckowian, Christopher M Adams, Md Shah A Siddique, Ashraful I Khan, Firdausi Qadri, Jason R Andrews, Mahmudur Rahman, Alfred M Spormann, Gary K Schoolnik, Allis Chien, Eric J Nelson

**Affiliations:** 1 Vincent Coates Foundation Mass Spectrometry Laboratory, School of Medicine, Stanford University, California; 2 Department of Civil and Environmental Engineering, School of Medicine, Stanford University, California; 3 Department of Pediatrics, School of Medicine, Stanford University, California; 4 Department of Microbiology, School of Medicine, Stanford University, California; 5 Department of Medicine, School of Medicine, Stanford University, California; 6 Department of Pediatrics, University of Florida, Gainesville; 7 Department of Environmental and Global Health, University of Florida, Gainesville; 8 Institute of Epidemiology, Disease Control, and Research, Ministry of Health and Family Welfare, Government of Bangladesh, Dhaka, Bangladesh; 9 Infectious Diseases Division, International Centre for Diarrhoeal Disease Research, Dhaka, Bangladesh

**Keywords:** Diarrhoea, diarrhea, cholera, mass spectrometry, antimicrobial resistance, AMR, LC/MS, *Vibrio cholerae*, Bangladesh

## Abstract

**Background:**

A first step to combating antimicrobial resistance in enteric pathogens is to establish an objective assessment of antibiotic exposure. Our goal was to develop and evaluate a liquid chromatography–ion trap mass spectrometry (LC/MS) method to determine antibiotic exposure in patients with cholera.

**Methods:**

A priority list for targeted LC/MS was generated from medication-vendor surveys in Bangladesh. A study of patients with and those without cholera was conducted to collect and analyze paired urine and stool samples.

**Results:**

Among 845 patients, 11% (90) were *Vibrio cholerae* positive; among these 90 patients, analysis of stool specimens revealed ≥1 antibiotic in 86% and ≥2 antibiotics in 52%. Among 44 patients with cholera and paired urine and stool specimens, ≥1 antibiotic was detected in 98% and ≥2 antibiotics were detected in 84%, despite 55% self-reporting medication use. Compared with LC/MS, a low-cost antimicrobial detection bioassay lacked a sufficient negative predictive value (10%; 95% confidence interval, 6%–16%). Detection of guideline-recommended antibiotics in stool specimens did (for azithromycin; *P* = .040) and did not (for ciprofloxacin) correlate with *V. cholerae* suppression. A nonrecommended antibiotic (metronidazole) was associated with decreases in anaerobes (ie, *Prevotella* organisms; *P* < .001).

**Conclusion:**

These findings suggest that there may be no true negative control group when attempting to account for antibiotic exposure in settings like those in this study.

Infectious diseases research on how antibiotics influence infection and transmission of pathogens relies heavily on self-reported medication use that is rarely objectively confirmed [1–3]. This problem leads to avoidable heterogeneity between control and experimental study arms and, potentially, to generation of cross-study conflicts. This is important for antimicrobial resistance (AMR) research because efforts to assess rates of AMR emergence under antibiotic pressure may be compromised by unknown antibiotic exposure. This problem impedes development of evidence-based guidelines that account for AMR emergence in the infected host and AMR transmission to the population.

We are specifically interested in diarrheal diseases because of the high rate of inappropriate use of antibiotics. One explanation is that most cases are caused by noninvasive bacterial pathogens or viral agents that require rehydration alone [4–6]. Antibiotics are typically restricted to invasive bacterial pathogens and select noninvasive pathogens, including *Vibrio cholerae* [6–8]. Despite guideline restriction, antibiotics are commonly sold in settings that have little regulatory oversight, such as Southeast Asia [9, 10]; this is likely one explanation for the high rate of AMR among enteric pathogens. For example, *V. cholerae* can be resistant to most antibiotic classes even though guidelines restrict treatment to macrolides, tetracyclines, fluoroquinolones, and trimethoprim/sulfamethoxazole [7, 11–15]. Bacterial taxa have been identified that are associated with early (for *Escherichia*, *Streptococcus*, and *Enterococcus* organisms), middle (for *Bacteroides* organisms), and late (for *Prevotella* and *Roseburia* organisms) phases of recovery from cholera [16]. Their resistance profiles are difficult to determine, given that some of these bacteria are fastidious. The degree to which inappropriate antibiotic use influences pathogenic and nonpathogenic microbiota has not been thoroughly studied, in part because of limitations in assessing antibiotic exposure.

Affordable and reliable assays to detect antibiotics in urine and stool samples are lacking [17–20]. One low-cost approach to determine whether a patient is excreting an antibiotic is to expose pan-susceptible bacteria to the patient’s urine and test for inhibition of growth. While this bioassay may indicate that an antimicrobial is present, it does not identify the agent and lacks sensitivity because the concentration must be sufficient to inhibit growth of the indicator organism [21, 22]. Liquid chromatography–mass spectrometry (LC/MS) enables high specificity and sensitivity for multiple analytes and has become tractable because instrumentation is more accessible and associated costs are decreasing [17, 18]. In this study, our objectives were to develop a scalable MS method that identifies clinically relevant antibiotics in diarrheal stool specimens, determines how widespread antibiotics are in patients with cholera, and identifies effects on the microbiota.

## METHODS

### Subjects

Samples were collected as part of a previously published study [23] that was approved by the institutional review boards at Stanford University School of Medicine (6208) and the Institute of Epidemiology, Disease Control, and Research, Bangladesh Ministry of Health and Family Welfare (IEDCR/IRB/2015/03). Participants provided written consent/assent.

### Pharmacy Survey

Medication vendors were surveyed in rural Bangladesh (Basta Union, Keraniganj Upazila, Dhaka District) to name and price the “three most common antibiotics used to treat diarrhea.”

### Clinical Study

The study was conducted from September to December 2015 at a district and subdistrict government hospital in rural Netrokona, Bangladesh. The sites were chosen because they are resource constrained with frequent diarrheal disease outbreaks [23, 24]. Inclusion criteria were an age of ≥2 months and the presence of acute diarrhea, defined as ≥3 loose stools in the past 24 hours and a total duration of <7 days. Patients with comorbidities were excluded.

### Sample Collection

The first urine and stool samples voided were collected before the administration of hospital antibiotics. Urine specimens had a volume of 1.5 mL and were stored at −20°C. Stool specimens had a volume of 2 mL; samples were placed in 6 mL of RNAlater (Invitrogen), stored on site at 4°C, and later frozen at −80°C. Stool samples were tested with a point-of-care cholera test (Crystal VC, Span Diagnostics). The first and last stool specimens collected each day and specimens testing positive for *V. cholerae* at the point of care were stored at 4°C in Cary Blair medium and transported to the central laboratory.

### Microbiological Analysis

#### Culture and Antibiotic Sensitivity Testing

Samples in Cary Blair medium underwent selective culture and sensitivity testing for *V. cholerae* at the IEDCR and the International Centre for Diarrhoeal Disease Research, Bangladesh [23, 25].

#### *Molecular Detection of* V. cholerae *and Vibriophages*

DNA from stool samples suspended in RNAlater was extracted using the MoBio PowerSoil 96-well kit. Each extraction batch contained *V. cholerae* resuspended in LB medium at concentrations relevant to those in stool specimens from patients with cholera (ie, 1 × 10^8^ and 5 × 10^8^ colony-forming units/mL). DNA extracts were screened for *V. cholerae* by 2 methods. The first approach consisted of a 384-well quantitative polymerase chain reaction (qPCR) format (Light Cycler, Roche) that was performed in duplicate, using established qPCR primers for *tcpA*^*set1*^ and a cycle threshold for positivity set to <29 [26]. The second approach involved a 5184 nanowell qPCR format (Tokara) with a 100-nL reaction volume, using *tcpA*^*set1*^ and *tcpA*^*set2*^ primers and a cycle threshold for positivity set to <28 (Supplementary Table 1). For both approaches, indeterminate results were assessed by PCR, using *ompW* primers [27]. Vibriophages (ICP1, ICP2, and ICP3) were identified by conventional PCR, using standard techniques (Supplementary Table 1); templates were the same as those used in the microbiota analysis (see below), and ICP1, ICP2, and ICP3 controls were provided by A. Camilli (Tufts University, Boston, MA).

#### Antimicrobial Detection in Urine Specimens by a Bioassay

Urine specimens were tested for antibiotics as previously described [21, 22]. A lawn of pan-susceptible *Kocuria rhizophila* (ATCC 9341) was made on sheep blood agar and Mueller-Hinton agar (Supplementary Figure 3) [18]. Ten microliters of urine supernatant was spotted onto disks in triplicate. The disks were dried, placed on the bacterial lawns, and incubated overnight at 37°C. Any zone of inhibition was scored as positive. Positive controls were urine specimens collected from a volunteer 5 hours after metronidazole (500 mg) and ciprofloxacin (750 mg) administration, as well as pure antibiotics in normal saline. Negative controls were urine specimens obtained from the same volunteer before antibiotic administration, as well as normal saline. Limits of detection for assays were derived from Clinical and Laboratory Standards Institute guidelines (Wayne, PA): 0.12 mg/mL amoxicillin, 0.12 mg/mL azithromycin, 0.115 mg/mL ceftriaxone, 2.5 μg/mL ciprofloxacin, and 0.47 mg/mL tetracycline.

**Table 1.** Frequency of Antibiotic Detection in Paired Urine and Stool Samples From Patients With Cholera

Antibiotic	Positivity, Specimens, No. (%)		Cohen κ (95% CI)^a^	McNemar’s χ ^2^ Test^a^	
	Urine	Stool		Statistic	*P*
Ciprofloxacin	35 (80)	30 (68)	0.71 (.48–.94)	3.2	.07
Metronidazole	34 (77)	26 (59)	0.60 (.36–.83)	6.13	.01
Azithromycin	14 (32)	13 (30)	0.41 (.12–.70)	0	>.999
Tetracycline	5 (11)	5 (11)	1 (NA)	NA	
Ceftriaxone	2 (5)	0 (0)	… ^b^	… ^b^	
Erythromycin	1 (2)	1 (2)	… ^b^	… ^b^	
Amoxicillin	1 (2)	0 (0)	… ^b^	… ^b^	

Abbreviation: NA, not applicable.

^a^For the comparison of stool and urine specimens.

^b^Fields with <5 medications detected were not analyzed.

#### Microbiota Analysis

Microbiota were profiled by preparing DNA with the QIAamp Fast DNA Stool Mini Kit (Qiagen) with bead beating, barcoded amplification of the V1 to V2 segment of the 16S ribosomal gene (Supplementary Table 1), and sequencing of pooled libraries with an Illumina MiSeq [28]. Operational taxonomic units were made with a threshold of 97% similarity and taxonomically mapped to the Greengenes 16S database [29]. Quality control and comparative analyses (eg, to determine α and β diversities) were performed without rarefaction, using standard methods within CLC Genomics Workbench v11.0 (Qiagen; Supplementary Materials).

### Sample Extraction Procedure for LC/MS

Protein was precipitated from urine samples with a 1:1 ratio (v/v) of cold methanol, sonicated, and centrifuged; supernatant was diluted with methanol and water (1:1 v/v) plus 0.1% formic acid; 10 μL was injected for LC/MS. Because of the high protein concentration in stool samples, extraction required additional steps. Supernatant was obtained by centrifugation and filtered (0.2 μM surfactant-free cellulose acetate; Thermo Scientific Nalgene). Protein was precipitated with a 1:7 ratio (v/v) of methanol; samples were sonicated and centrifuged. Both urine and stool supernatants were diluted with methanol and water (1:1 v/v) plus 0.1% formic acid; 10 μL was injected for LC/MS.

### LC/MS Method for Detection of Clinically Relevant Medications

The LC/MS method represents a qualitative approach and uses ion trap MS, rather than triple-quadrupole targeted MS, because the former enables both the screening of targeted analytes and identification of untargeted compounds, such as major metabolites. The unified LC/MS method used an 1100 series high-performance liquid chromatography system (Agilent Technologies) integrated with an LTQ XL ion trap mass spectrometer (Thermo Fisher Scientific). The mass spectrometer acquired both full scan (m/z 130 -1000) and MS2 data using heated electrospray ionization (HESI) in positive mode. To enhance identification of parent compounds and metabolites, data dependent acquisition in dynamic exclusion mode was utilized. LC was performed on a 2.1 × 150–mm Hypersil Gold aQ column (particle size, 3 μm), with gradient elution; the flow rate was 0.25 mL/minute, and the column temperature was 40°C. Mobile phases were 0.1% formic acid in water (A) and 0.1% formic acid in acetonitrile (B). The gradient elution profile was as follows: an initial hold at 0% B for 1 minute, followed by a linear gradient of 0%–95% B for 15 minutes, and then a hold at 95% B for 1 minute before equilibrating back to 0% B; the total run time was 20 minutes. Controls included blank samples run intermittently during analysis.

Analytes were identified by the retention time, mass-to-charge ratio, and fragmentation data. The analytical response, measured as a chromatographic peak area, was determined for each analyte. Analytes with peak areas of >1 × 10^5^ were designated “major,” and those with peak areas of <1 × 10^5^ were designated “minor.” LC/MS sensitivity was determined by spiking 16 analytes into an extracted blank urine matrix, followed by serial dilutions. Dilutions ranged from 2 μg/mL to 0.512 ng/mL. Limits of detection were determined based on sufficient peak area at the correct retention time: peak area values >0.4 × 10^5^ were considered valid for analyte confirmation. Owing to the diverse structures of analytes and ionization efficiencies, detection limits ranged widely. Limits of detection in the blank urine matrix corresponded to the following serial dilutions: 1.28 ng/mL for trimethoprim and ondansetron, 3.2 ng/mL for azithromycin and erythromycin, 8 ng/mL for ciprofloxacin, 20 ng/mL for sulfamethoxazole and doxycycline, 50 ng/mL for metronidazole, amoxicillin, tetracycline, and omeprazole, 125 ng/mL for paracetamol, ceftriaxone, and furazolidone, and 320 ng/mL for cephalexin and penicillin.

### Statistical Analysis

The Cohen κ coefficient was used to assess interrater agreement between results of urine and stool assays. The McNemar χ ^2^ test was used to test marginal frequencies (null hypothesis). Analyses were performed in GraphPad Prism 8.0.1 and R v3.4.1/RStudio v1.1.0153 [30]. Permutational multivariate analysis of variance analysis was conducted with CLC Genomics Workbench v11.0 (Qiagen).

## RESULTS

### Antibiotic Survey of Medication Vendors

**Figure 1.**
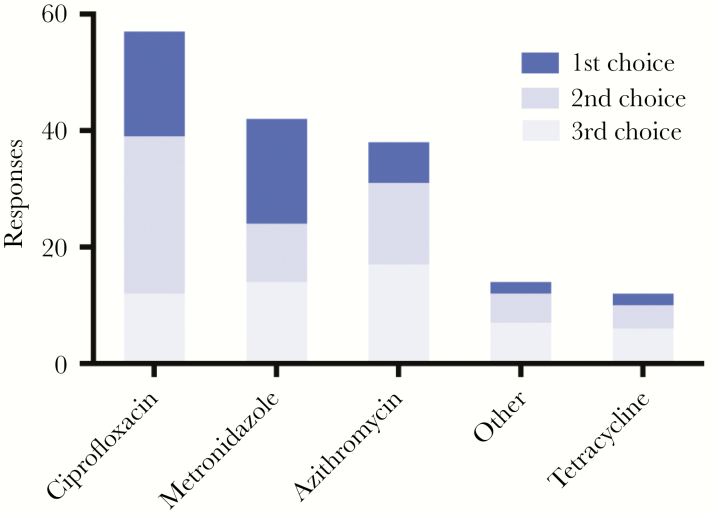
Responses to the question “Name the top three antibiotics you sell for the treatment of diarrheal disease” among 62 rural medication vendors. “Other” refers to amoxicillin (1 respondents; median cost, $0.06/tablet), ampicillin (1; $0.06/tablet), erythromycin (5; $0.10/tablet or $0.76/liquid dose), and nitazoxanide, an antiparasitic medication (3; $0.13/tablet). Pricing for ciprofloxacin, metronidazole, azithromycin, is tetracycline are provided in the text.

Responses from 62 medication vendors to the question “Name the top three antibiotics you sell for the treatment of diarrheal disease” were ciprofloxacin (57 respondents; median cost, $0.19/tablet), metronidazole (55; $0.02/tablet), azithromycin (38; $0.44/tablet), tetracycline (13; $0.03/tablet), and “other” (10; Figure 1). The survey, World Health Organization (WHO) guidelines on diarrheal disease treatment, and WHO guidelines for treatment of acute respiratory infection (patients with diarrhea often have concomitant pneumonia) were used to generate the following list of antibiotics for targeted LC/MS: azithromycin, amoxicillin, cephalexin, ceftriaxone, ciprofloxacin, doxycycline, erythromycin, furazolidone, metronidazole, penicillin V, sulfamethoxazole/trimethoprim, and tetracycline.

### Antibiotic Detection by LC/MS

#### Sample Collection and Preparation

Stool samples were collected from 845 of 961 enrolled patients. PCR identified 90 patients with cholera, among whom the median age was 24 years (range, 0.5–70 years), and the sex of 50% was female. Ten paired samples from patients without cholera were analyzed.

#### Detection of Antibiotics

**Figure 2.**
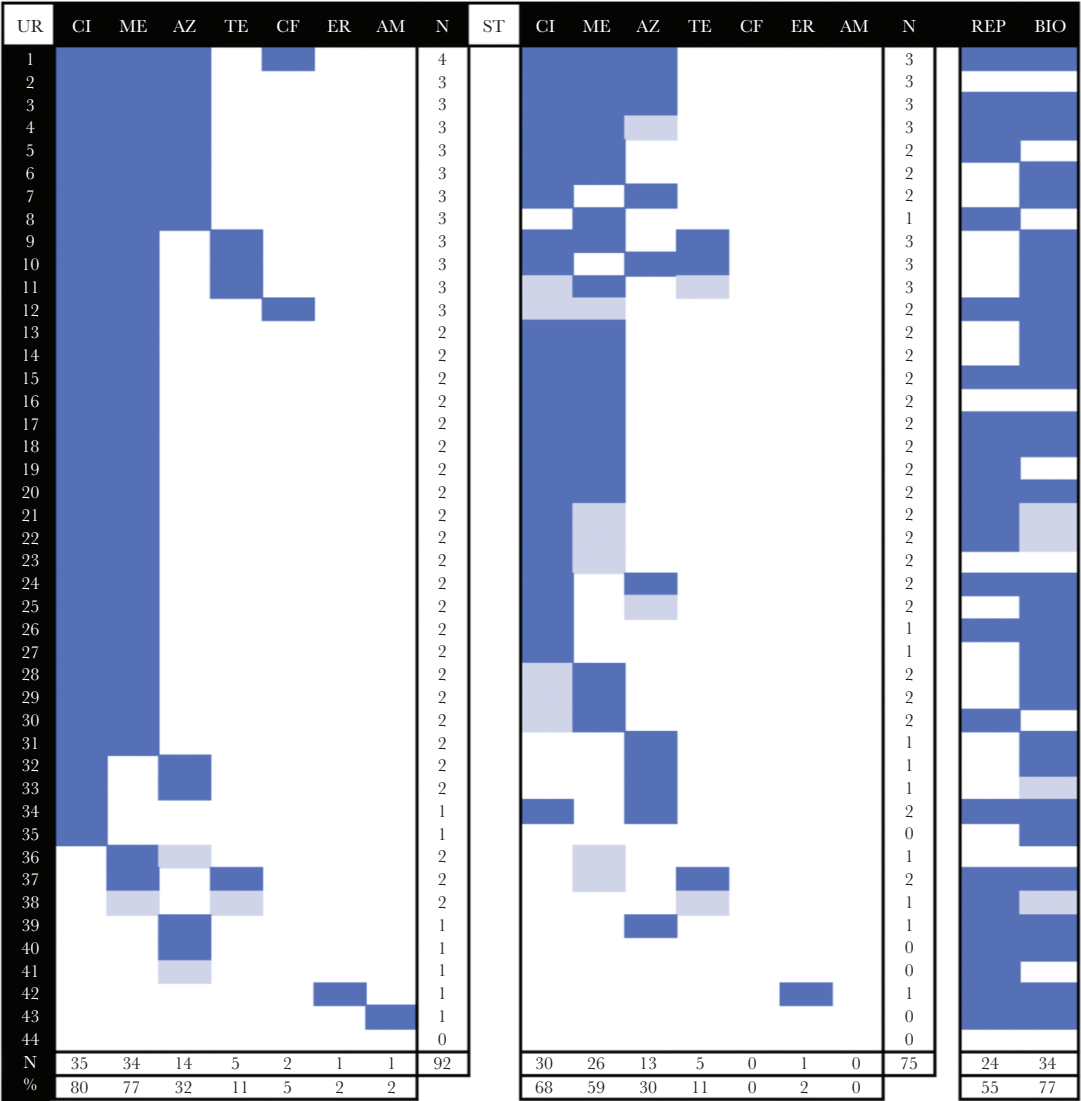
Frequency of antibiotic detection in paired urine (UR; left) and stool (ST; right) samples from patients with cholera, using liquid chromatography/mass spectrometry, self-reported prior medication use (REP), and bioassay with urine extracts (BIO). Dark blue denotes positive results or self-report, light blue denotes trace detection, and white denotes negative results or no self-report. For urine and stool specimens, the number of antibiotics detected per sample is shown at right, and the number of times (%) an antibiotic was detected across samples is shown at the bottom. AM, amoxicillin; AZ, azithromycin; CF, ceftriaxone; CI, ciprofloxacin; ER, erythromycin; ME, metronidazole, TE, tetracycline.

**Figure 3.**
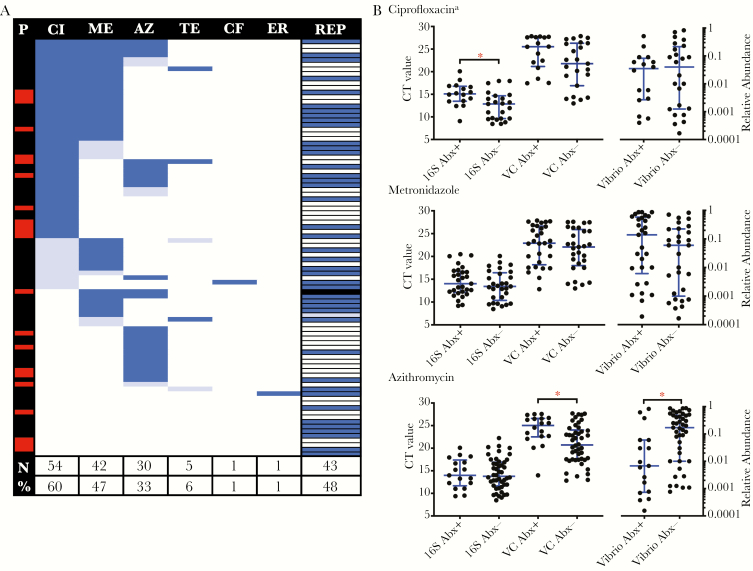
Frequency of antibiotic detection in stool specimens from patients with cholera and impact on *Vibrio cholerae*. *A*, Frequency of antibiotic detection by liquid chromatography/mass spectrometry and self-report (REP) among all patients with cholera from whom stool specimens were collected. The number of times (%) an antibiotic was detected across samples is shown at the bottom. The red lines at left denote positivity for phage, and the black line at right denotes an unknown history. Dark blue, positive result or self-report; light blue, trace detection; white, negative result or no self-report. *B*, Paired comparisons of total bacteria (16S ribosomal DNA) and *V. cholerae* by nanoliter quantitative polymerase chain reaction analysis, and the relative abundance of *Vibrio* species with (Abx+) and without (Abx-) detection of the specified antibiotic. Bars and whiskers denote medians and interquartile ranges, respectively. Analyses were restricted to samples without detection of trace antibiotic and phage. See Supplementary Table 2 for further details. AM, amoxicillin; AZ, azithromycin; CF, ceftriaxone; CI, ciprofloxacin; CT, cycle threshold; ER, erythromycin; ME, metronidazole, TE, tetracycline. *P < .05, by the Mann-Whitney *U* test, with adjustment for multiple comparisons. ^a^Samples negative for ciprofloxacin were also negative for metronidazole.

Extracted ion chromatograms for the antibiotic control mixture had consistent elution times and enabled identification of targets in paired urine and stool specimens (Supplementary Figures 1 and 2); positive and negative control urine samples were consistent. LC/MS of paired stool and urine specimens from patients with cholera found ≥1 antibiotic in 98% (43 of 44) and ≥2 antibiotics in 84% (37 of 44). A total of 92 antibiotics (mean, 2.1 antibiotics/patient; range, 0–4 antibiotics/patient) and 75 antibiotics (mean, 1.7 antibiotics/patient; range, 0–3 antibiotics/patient) were identified in 44 paired urine and stool samples, respectively (Figure 2). Analysis of all stool samples identified ≥1 antibiotic in 86% (77 of 90) and ≥2 antibiotics in 52% (47 of 90; Figure 3A), for a total of 133 observations (mean, 1.5 antibiotic/patient; range, 0–3 antibiotics/patient). Results for 10 patients without cholera were similar (Supplementary Figure 4).

Frequencies of ciprofloxacin and tetracycline detection from patients with cholera were not statistically different in paired urine and stool specimens (Table 1). The Cohen κ for correlation of metronidazole detection in urine and stool specimens was 0.60 (95% confidence interval [CI], .36–.83), and the difference was significant (*P* = .01); there was no correlation between azithromycin detection in urine and stool specimens. First-pass metabolism and degradation in the gastrointestinal tract influenced differential detection in urine and stool specimens. Detection of metabolites provided additional confirmation of the presence of the investigated medications.

#### Nonantibiotic Detection

Among paired samples from patients with cholera, ondansetron and metabolites were detected in urine alone in 48% (21 of 44) [31]. Paracetamol was detected in 11% of urine samples (5 of 44) and in 0.2% of stool specimens (1 of 44). In patients without cholera, paracetamol was detected in 5 of 6 urine specimens and 6 of 10 stool specimens. Omeprazole was not detected.

### Antimicrobial Detection by a Bioassay

The bioassay found that 77% of urine samples (34 of 44) inhibited growth of *K. rhizophila* (Supplementary Figure 3). Compared with LC/MS, the low-cost antimicrobial activity assay had a sensitivity of 79% (95% CI, 64%–90%), a specificity of 100% (95% CI, 2.5%–100%), a positive predictive value of 100% (95% CI, not applicable), and a negative predictive value of 10% (95% CI, 6%–17%). Similar patterns of antimicrobial activity were observed in patients without cholera (Supplementary Figure 4).

### Antibiotic Susceptibility Profile of *V. cholerae* Isolates

Among patients from whom specimens were collected and cultured, 6.4% (16 of 249) were positive for *V. cholerae*. All isolates were susceptible to ciprofloxacin despite detection of ciprofloxacin by LC/MS in 5 of 7 available urine samples and 8 of 16 stool samples. All isolates (100% [16 of 16]) were azithromycin susceptible. Intermediate resistance was present for trimethoprim/sulfamethoxazole (81% [13 of 16]) and tetracycline (75% [12 of 16]). All isolates (100% [16 of 16]) were erythromycin resistant.

### Medication Detection by LC/MS Versus Self-reported Use

Patients were asked whether medications had been taken for their current illness before admission, because they were unable to articulate antibiotic names. Among patients with paired urine and stool samples, 55% (24 of 44) reported taking medication before admission. There was no concordance between self-reported medication use and detection of medication by LC/MS (Cohen κ, 0.054); among all patients with cholera, 48% (43 of 90) reported medication use (Figure 3A). Findings were similar for patients without cholera (Supplementary Figure 4).

### Microbiota Correlates of Antibiotic Exposure

**Figure 4.**
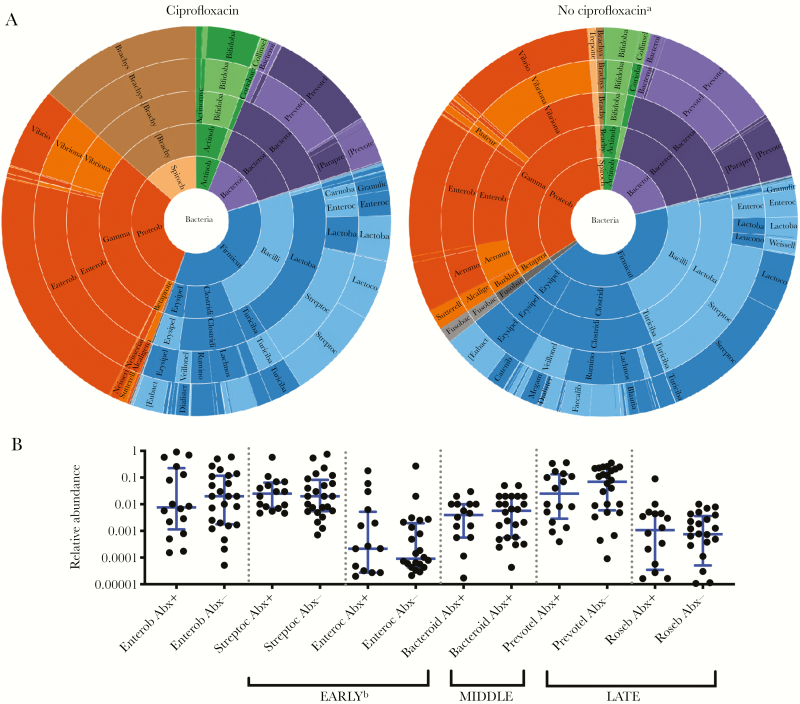
Ciprofloxacin is associated with minimal microbiota changes. *A*, Comparison of stool samples from patients with cholera in which ciprofloxacin was or was not detected; samples with phage or trace antibiotic detection were removed. *P* = .158, by permutational multivariate analysis of variance (Bray-Curtis dissimilarities). *Brachyspira* species are in brown. *B*, Relative abundance of taxa previously shown to be associated with early, middle, and late phases of recovery from cholera, with (Abx+) and without (Abx-) antibiotic detection. No significant differences were detected by the Mann-Whitney *U* test (α = 0.05). *P* values were adjusted for multiple comparisons. Bars and whiskers denote medians and interquartile ranges, respectively. Analyses were restricted to samples without detection of trace antibiotic and phages. See Supplementary Table 2 for further details. Bacteroid, *Bacteroides*; Enterob, Enterobacteriaceae (family); Enteroc, *Enterococci*; Prevotel, *Prevotella*; Roseb, *Roseburia*; Streptoc, *Streptococci*. ^a^Samples negative for ciprofloxacin were also negative for metronidazole. ^b^Enterobacteriaceae is grouped with genera associated with the early phase of recovery because the family contains *Escherichia,* which is known to be associated with this phase.

**Figure 5.**
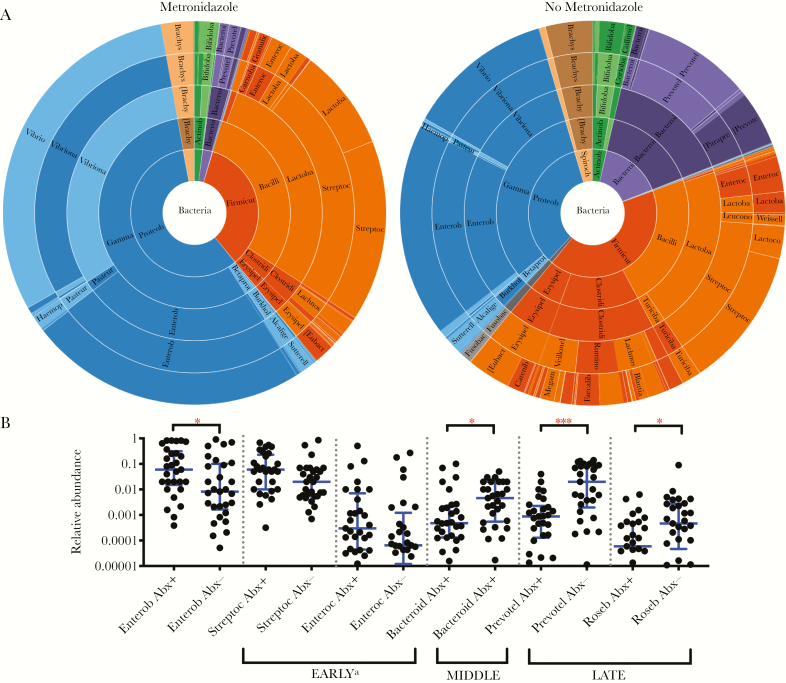
Metronidazole is associated with relative decreases in anaerobes, including *Prevotella* species. *A*, Comparison of stool samples from patients with cholera in which metronidazole was or was not detected; samples with phage or trace antibiotic detection were removed. *P* = .001, by permutational multivariate analysis of variance (Bray-Curtis dissimilarities). *Prevotella* species are in purple. *B*, Relative abundance of taxa previously shown to be associated with early, middle, and late phases of recovery from cholera, with (Abx+) and without (Abx-) antibiotic detection. Bars and whiskers denote medians and interquartile ranges, respectively. Analyses were restricted to samples without detection of trace antibiotic and phage. See Supplementary Table 2 for further details. *P < .05 and ***P < .001, by the Mann-Whitney *U* test, with adjustment for multiple comparisons. Bacteroid, *Bacteroides*; Enterob, Enterobacteriaceae (family); Enteroc, *Enterococci*; Prevotel, *Prevotella*; Roseb, *Roseburia*; Streptoc, *Streptococci*.

**Figure 6.**
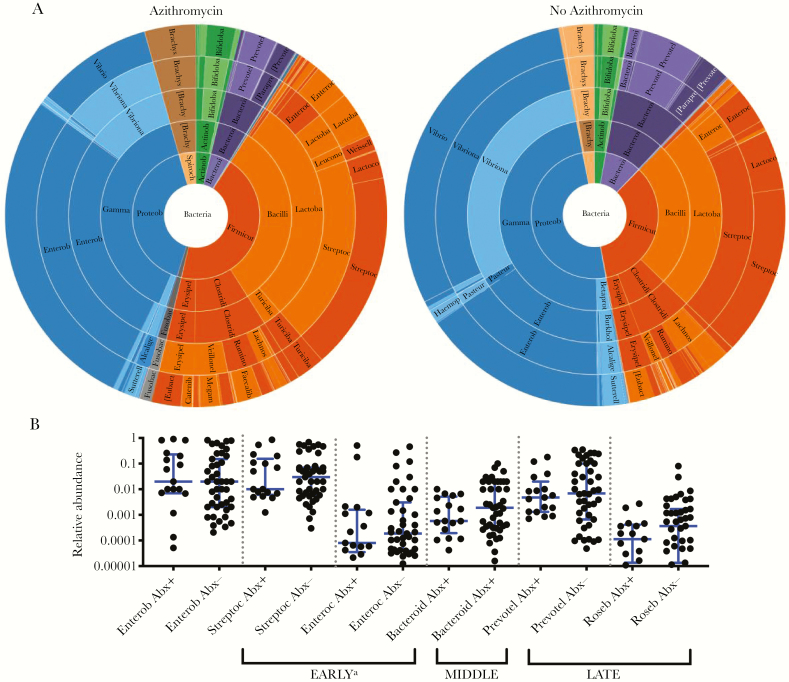
Azithromycin is not associated with major taxonomic changes other than suppression of *Vibrio cholerae*. *A*, Comparison of stool samples from patients with cholera in which azithromycin was or was not detected; samples with phage or trace antibiotic detection were removed. *P* = .041, by permutational multivariate analysis of variance (Bray-Curtis dissimilarities). *Vibrio* species are in blue. *B*, Relative abundance of taxa previously shown to be associated with early, middle, and late phases of recovery from cholera, with (Abx+) and without (Abx-) antibiotic detection. No significant differences were detected by the Mann-Whitney *U* test (α = 0.05). *P* values were adjusted for multiple comparisons. Bars and whiskers denote medians and interquartile ranges, respectively. Analyses were restricted to samples without detection of trace antibiotic and phage. See Supplementary Table 2 for further details. Bacteroid, *Bacteroides*; Enterob, Enterobacteriaceae (family); Enteroc, *Enterococci*; Prevotel, *Prevotella*; Roseb, *Roseburia*; Streptoc, *Streptococci*. ^a^Enterobacteriaceae is grouped with genera associated with the early phase of recovery because the family contains *Escherichia,* which is known to be associated with this phase.

Given that only 1 sample was negative for antibiotics as measured by analysis of paired urine and stool specimens, stool samples were analyzed for correlates of microbiota changes as a function of antibiotic detection in stool alone. Samples with lytic vibriophage or trace antibiotic detection were removed as potential confounders. Permutational multivariate analysis of variance (based on Bray-Curtis dissimilarities) was used to assess β diversity as a function of (1) detection (n = 59) versus nondetection (n = 9) of any antibiotic (pseudo F statistic, 1.56; *P* = .057; Supplementary Table 2); (2) detection (n = 29) versus nondetection (n = 24) of metronidazole (pseudo F statistic, 2.78; *P* = .001; Figure 5A); (3) among metronidazole negative samples, detection (n = 16) versus nondetection (n = 24) of ciprofloxacin (pseudo F statistic, 1.23; *P* = .158; Figure 4A); and (4) detection (n = 17) versus nondetection (n = 43) of azithromycin (pseudo F statistic, 1.71; *P* = .041; Figure 6A). Bivariate comparisons of α diversity by antibiotic exposure were not significantly different (*P* > .05, by the Mann-Whitney *U* test; Supplementary Figure 5).

The relative abundances at the level of genera were analyzed in bivariate comparisons as a function of the presence of any antibiotic, ciprofloxacin, metronidazole, and azithromycin (Figures 3–6 and Supplementary Table 2). Samples with trace detection and those harboring lytic vibriophages were removed as potential confounders (Figure 3A). By nanoliter qPCR (Figure 3B and Supplementary Table 2), a decrease in bacterial load correlated with ciprofloxacin detection (*P* = .009) and a decrease in *V. cholerae* load was associated with azithromycin (*P* = .042). Comparisons of the relative abundance revealed that metronidazole was associated with a decrease in *Prevotella* organisms (*P* < .001), which are associated with patients who recovered from cholera and healthy controls [16]. In contrast to the relative decreases of anaerobes (eg, *Prevotella*, *Bacteroides*, *Blautia*, and *Ruminococcus* organisms), metronidazole was associated with an increase in taxa associated with active cholera and/or the early phase of recovery from cholera (ie, Enterobacteriaceae; Supplementary Table 2). Ciprofloxacin was associated with a marginal increase in the relative abundance of *Brachyspira* organisms (Supplementary Table 2); species of this genus have been characterized as pathogens of both humans and livestock [32, 33].

## DISCUSSION

The objective of this study was to determine how widespread antibiotic use is among patients with cholera and to test the concordance of these findings with patients’ self-reported history of medication use. LC/MS analysis of paired urine and stool specimens from patients with cholera found ≥1 antibiotic in 98% despite only 55% reporting any medication use. While antibiotic detection correlated with anticipated shifts in the microbiome and pathogen abundance, the study identified negative consequences of an ineffective antibiotic on taxa previously found associated with cholera recovery and healthy controls. These findings represent a call to awareness that antibiotic exposure is widespread and discordant with self-reported patient history. Given the potential impact on the microbiota, studies at high risk of confounding by unknown exposure to antibiotics will benefit from LC/MS analysis.

The discordance may be explained by respondent confusion due to cultural, economic, and educational challenges. Patient perception of secondary gain by answering questions falsely was minimized by hiring professional data collectors dressed in nonprovider uniforms and providing consent procedures that stated that medical care would not be affected by enrollment. Unintentional antibiotic exposure is possible, given that high levels of antibiotics can be found in environmental sources, including commercial food products [7].

Rural medication vendors were surveyed to identify medications sold for diarrheal disease. The intent was to assess prescription habits and cost and to prioritize medications for targeted LC/MS analysis. Ciprofloxacin and metronidazole were the 2 most common prescribed medications. Azithromycin, commonly used for respiratory infections, is the recommended treatment for *V. cholerae* resistant to other recommended antibiotics [5, 7] and the third most common antibiotic named for diarrhea treatment. Amoxicillin and penicillin V (which are used for respiratory tract infections) were included because diarrheal patients may have respiratory disease [34–36].

We hypothesized that antibiotic-resistant pathogens would be shed by patients shedding the corresponding antibiotic. Although susceptibilities to all antibiotics were not tested, all *V. cholerae* isolates were susceptible to ciprofloxacin, even though ciprofloxacin was detected in half of the isolate stool supernatants. These results were not anticipated, given that ciprofloxacin resistance is common among *V. cholerae* isolates in Bangladesh [37–39]. Explanations include protective niches [40], resistance mechanisms that do not recapitulate *in vitro* [41–43], limitations with antimicrobial susceptibility tests, and/or subinhibitory levels of antibiotic exposure.

Findings of LC/MS were consistent. An artificial control mixture containing standards of all analytes was run with each batch and showed consistent elution times. Blank samples distributed throughout each run did not show the presence of analytes. Chromatographic separation was designed to detect both parent medications and their metabolites, which are generally more polar than the parent drug and therefore elute earlier. The method monitored full scan (MS), MS^2^, and MS^3^. For analytes with the same molecular weight (eg, doxycycline and tetracycline), baseline chromatographic separation enabled unambiguous assignment. For analytes with complex metabolites (eg, azithromycin), MS fragmentation data aided in metabolite identification and therefore parent confirmation.

Different frequencies of detection between stool and urine specimens for some antimicrobials (eg, azithromycin) were likely attributed to differences in sample collection time and conditions, matrix complexity, and recovery. When the 16-component standard mix was spiked into a stool sample, 50% analyte loss was observed in filtered samples, compared with the nonfiltered samples. Further optimizing stool preparation to minimize dilution and avoid filtration may increase sensitivity. Differences in excretion pathways, possible first-pass metabolism, and degradation may also affect detection frequencies.

LC/MS poses benefit versus cost challenges. The advantages are that LC/MS identifies the antibiotic and has a higher sensitivity. In contrast, the bioassay offers a high sensitivity, specificity, and positive predictive value, but the negative predictive value is unacceptably low. The bioassay is tractable and less expensive, at $1–$2 per sample, compared with LC/MS (approximately $20–$160 per sample); however, the downstream cost of measurement bias without LC/MS is likely high.

The value of incorporating the LC/MS metadata into the microbiota analysis was 3-fold. First, it provided confirmation that metronidazole was associated with a relative reduction of expected taxa, such as anaerobic genera. In addition, the relative decrease of genera associated with disease recovery (eg, *Prevotella* spp.) provided an important example of the unanticipated consequences of inappropriate antibiotic use. Second, it supported azithromycin as a recommended agent for cholera, because of the reduction in the absolute and relative abundance of *V. cholerae*. Third, it revealed the lack of ciprofloxacin-associated suppression of *V. cholerae* despite *in vitro* detection of susceptibility by antimicrobial susceptibility testing. These data caution against the use of ciprofloxacin for cholera.

These findings should be viewed within the context of the limitations of the study. First, the study was conducted at only 2 sites within a single district in rural Bangladesh. Second, the mass spectrometry results are presented as qualitative data. LC/MS can provide quantitative data. We decided on a qualitative method not only due to cost and logistical constraints, but also to enable analyte annotation and detection of metabolites, and because physiologic variation in free water volume in urine and diarrheal stool may devalue quantitation. Third, the medication-vendor survey and clinical study were in different districts, yet both were in rural settings. Fourth, the microbiota analysis was restricted by a small sample size and limited antibiotic-negative stool samples. This limitation negated the ability to adjust for age and sex, resulted in a lack of significance for some bivariate comparisons after adjustment for false-discovery rates, and caused insufficient representation of important taxa, such as *Blautia obeum* and *Paracoccus aminovorans*, that are known to be associated with inibition *in vivo* and promotion of growth *in vitro* of *V. cholerae*, respectively [3, 44]. The V1-V2 primers did separate *Escherichia* organisms from other genera within the Enterobacteriaceae family. Enterobacteriaceae was grouped next to genera associated with the early phase of recovery from cholera, based published data on *Escherichia* species [16]. Despite these limitations, the methods represent a robust approach for monitoring antibiotic exposure that provide important correlates to assess antibiotic-associated host and microbiota phenomena.

The findings suggest that there may be no true negative control when attempting to account for antibiotic exposure in settings like those in this study. Similar studies that incorporate LC/MS will contribute to a more complete understanding of the scale of antibiotic exposure. Collectively, these data will provide important clinical insights that will help guide our approach to combating AMR and encouraging better antimicrobial stewardship.

## SUPPLEMENTARY DATA

Supplementary materials are available at *The Journal of Infectious Diseases* online. Consisting of data provided by the authors to benefit the reader, the posted materials are not copyedited and are the sole responsibility of the authors, so questions or comments should be addressed to the corresponding author.

jiz299_suppl_Supplementary_Data

## Notes

Notes

***Acknowledgments***. We thank the patients, for participating in this study; the diligent clinical and research team who made this study possible; L. Kahana, S. Kache, R. Blankenberg, C. Sarnquist, M. Barry, S. Luby, and Y. Maldonado, for their support at Stanford University; G. Morris, S. Rivkees, R. Autrey, K. Berquist, and M. Gaddy at the Emerging Pathogens Institute at the University of Florida, for administrative support and scientific guidance; A. Camilli at Tufts Univesity for reagents and mentorship, and V. Mai and M. Ukhanova, for their technical guidance and training in microbiome sample preparation and sequencing.

***Disclaimer.*** These funders had no role in study design, data collection and analysis, decision to publish, or preparation of the manuscript.

***Financial support.*** This work was supported by the National Institutes of Health (grant DP5OD019893 to E. J. N.); the University of Florida; and the Center for Innovation in Global Health, the Translational and Applied Medicine Center, and the Child Health Research Institute, Stanford University.

***Potential conflicts of interest.*** All authors: No reported conflicts.
