# Supplementary material for: Identification of Widespread Antibiotic Exposure in Patients With Cholera Correlates With Clinically Relevant Microbiota Changes
Source: J Infect Dis. 2019 Jun 13;220(10):1655–66. doi: 10.1093/infdis/jiz299 (PMC6782107; doi:10.1093/infdis/jiz299)
Supplement: jiz299_suppl_Supplementary_Data [file jiz299_suppl_supplementary_data.pdf]

SUPPLEMENTAL DATA

FIGURE S1

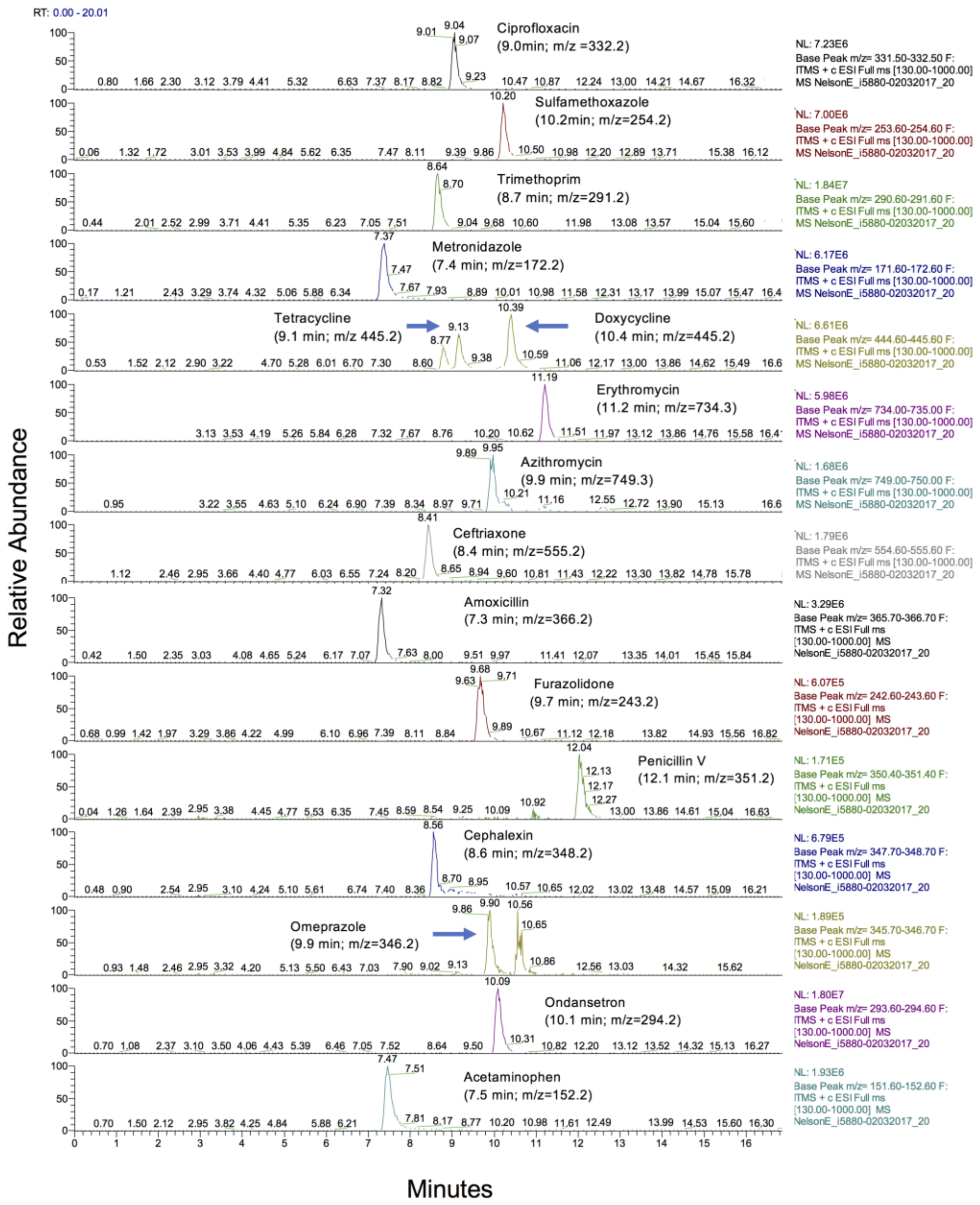

Figure S1. Representative extracted ion chromatograms (EICs) of control antibiotic mixture showing 16 analytes. X axis is the retention time (minutes) and Y is the relative abundance for each analyte; the scale is normalized to the largest peak in the selected time range. Doxycycline and tetracycline are displayed in the same EIC because the mass-to-charge ratio for each analyte is the same. Control antibiotic mixture was used to establish retention time and MS/MS data for analyte confirmation during sample analysis. Mass to charge ratios are included with the analyte label.

FIGURE S2.

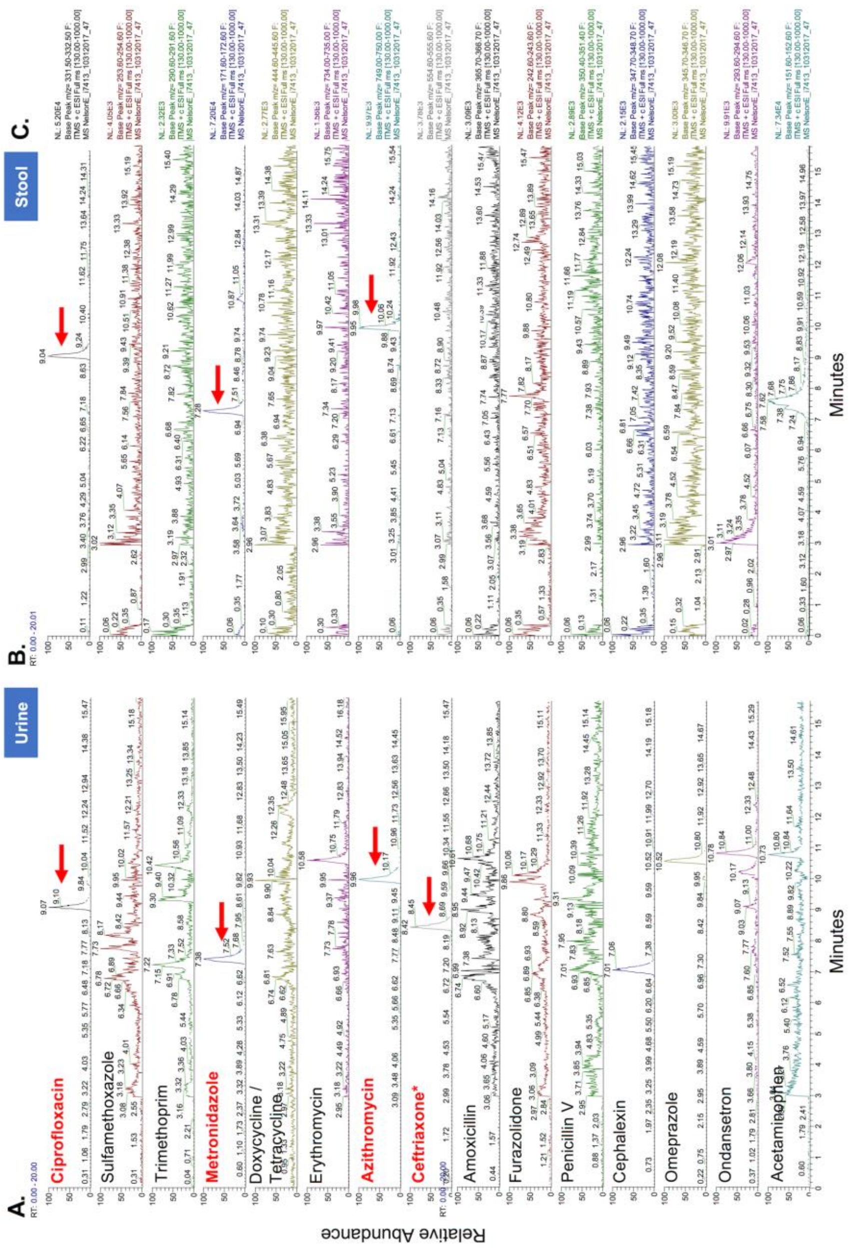

Figure S2. Representative extracted ion chromatograms (EICs) of urine and cholera stool. LC/MS ion extracted chromatograms (EICs) for 16 targeted analytes (labeled on right) for a paired urine sample (**A)** and stool sample (**B**) from a patient positive for *V. cholerae* by PCR but negative by culture. X axis is the retention time (minutes) and Y is the relative abundance for each analyte; the scale is normalized to the largest peak in the selected time range. Identity of analytes was confirmed by retention time and MS/MS data. **(C)** Intensity, base peak mass-to-charge ratio range, MS acquisition range.

FIGURE S3

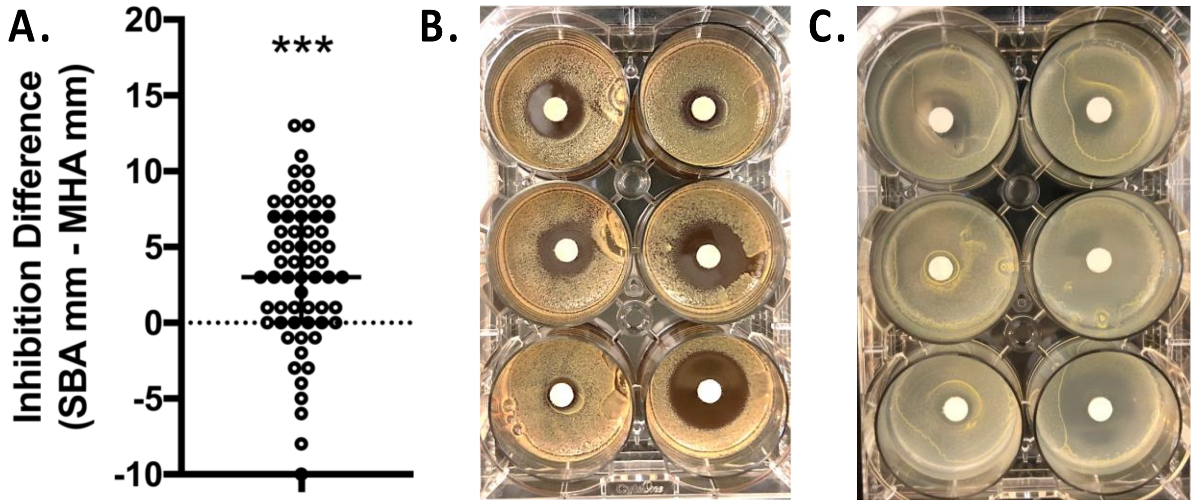

Figure S3. Antimicrobial detection in urine samples by biologic assay. (A) The distribution of the difference of the median diameter of the zone of inhibition of *Kocuria rhizophila* from cholera patient urine supernatants; N=60 samples positive for activity; controls not shown. Median and interquartile range shown. The analysis is derived from three independent assays on Sheep Blood Agar (SBA; **B**) versus Mueller-Hinton Agar (MHA; **C**); the Wilcoxon Signed-Rank statistic of 1341 was significant at p<0.0001 (***)

FIGURE S4

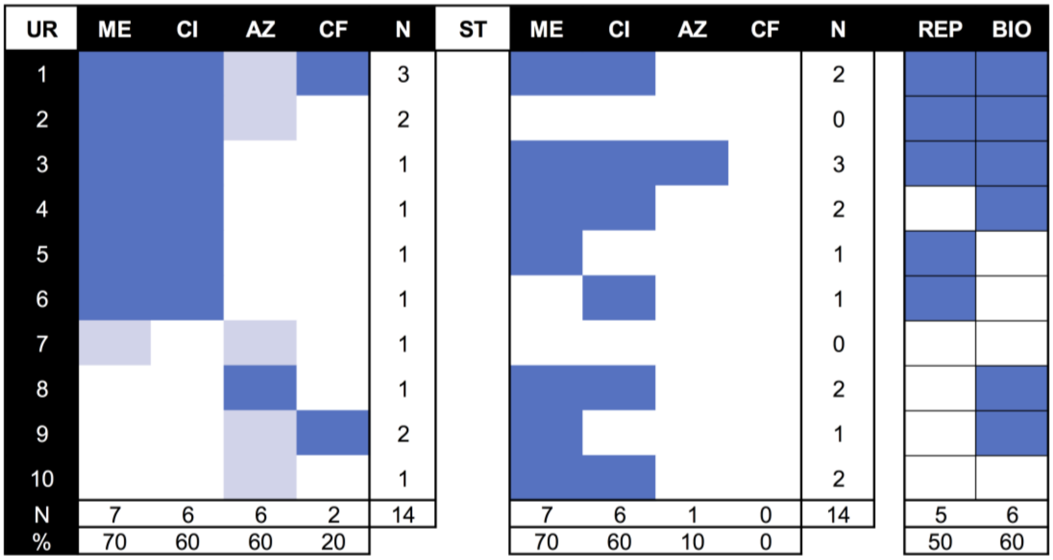

Figure S4. Frequency of antibiotic detection in paired urine and stool samples from non-cholera patients. Frequency of antibiotic detection by LC/MS of paired cholera patient urine (UR; left) and stool (ST; right), prior self-reported medication use (Rep) and biologic inhibition test with urine extract (Bio). Dark blue = positive, light blue = trace, and white = negative. Total antibiotic number is enumerated by column and row with percentage positive detection under totals. Trace amounts are considered as a positive detection and included in percentage calculations. CI= ciprofloxacin, ME=metronidazole, AZ=azithromycin, CF=ceftriaxone.

Figure S5

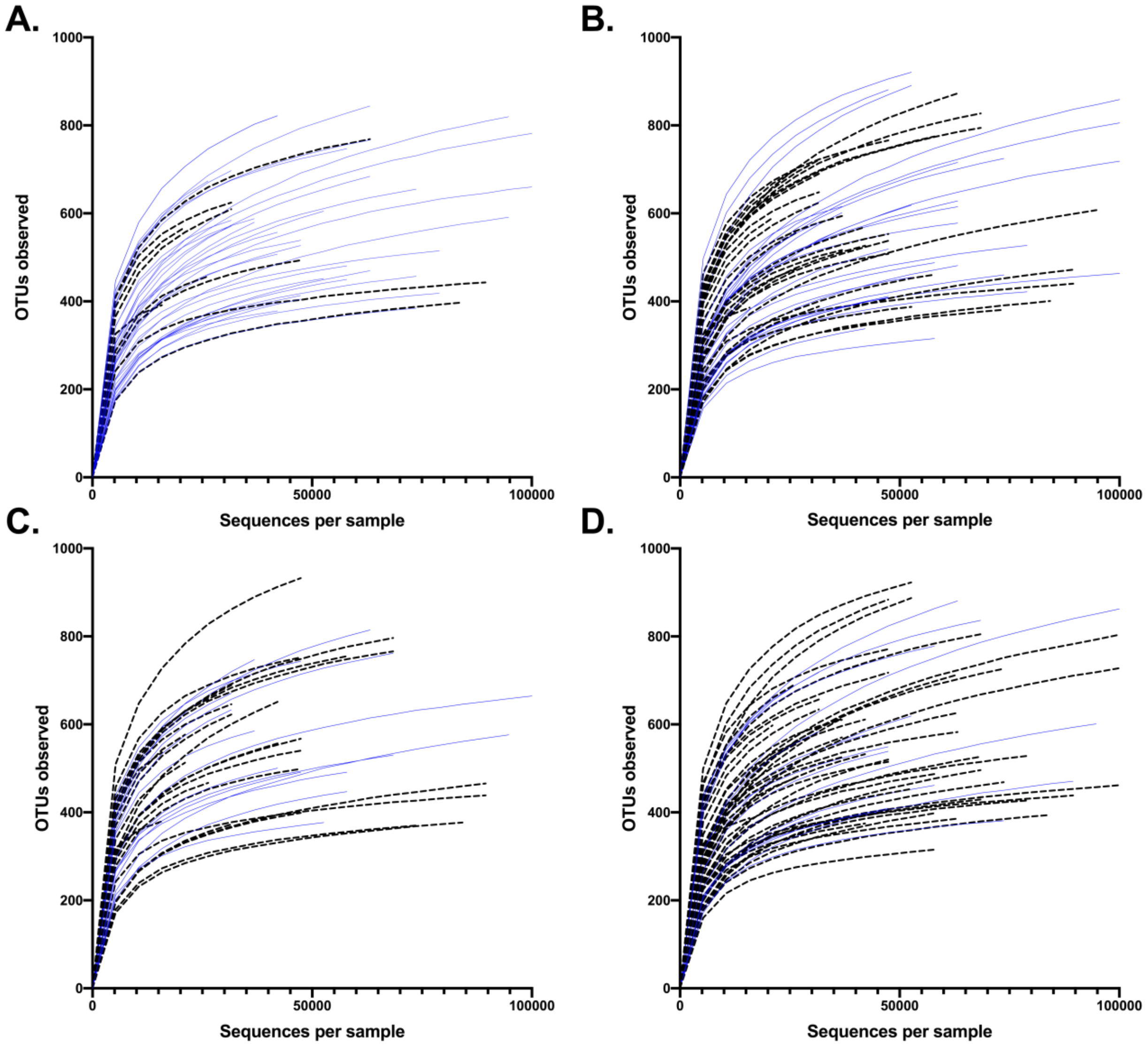

Figure S5. Bacterial diversity depicted by operational taxonomic unit (97% identify) and sequence number per sample for the following conditions: A. Any antibiotic vs no antibiotic detected. **B.** Metronidazole vs no metronidazole detected. **C.** Ciprofloxacin vs no ciprofloxacin/ no metronidazole detected. **D.** Azithromycin vs no azithromycin detected. Blue solid lines = antibiotic detected. Black dotted lines = did not contain the antibiotic. Samples with trace detection of the targeted antibiotic were removed. Data are derived from samples that did not harbor lytic vibriophages as tested by PCR. The alpha diversity for each bivariate comparison (panels A-D) was analyzed by the Mann-Whitney U test (two-tailed) at 15,000 and 30,000 sequences per sample. There was no difference in alpha diversity for each comparison ($p > 0.05$).

TABLE S1.

Table S1. Molecular reagents

Target	Primer Name	Sequence 5’ – 3’	Reference
ompW	ompW_F	CACCAAGAAGGTGACTTTATTGTG	Nandi et al.[27]
	ompW_R	GAACTTATAACCACCCGCG	
tcpA	*tcpA*^{set1}_F	ACTAAGGCTGCGCAAAATCT	Grembi & Spormann[26]
	tcpA^{set1}_R	GCCTCATCAGCTGAAACCTT	
tcpA	*tcpA*^{set2}_F	ACACGATAAGAAAACCGGTCA	Grembi & Spormann[26]
	tcpA^{set2}_R	GCCTTGGTCATATTCTGCGA	
ICP1	ICP1gp58F	AACGCTGCTTTTCCTTTTGA	Seed et al.[46]
	ICP1gp58R	CCCAGCATTGAGGACACTT	
ICP2	ICP2_4F	CGCTAGTTCTGGCAGTGA GT	This study
	ICP2_4R	TCCGTTCCAGTTCCAACAGG	
ICP2	ICP2_24R	AGAAGTCGCAAACGGGGTAC	This study
	ICP2_24R	AACGTGGTTCTCGTGAGTGG	
ICP3	ICP3gp5F	ATTGTCGAGTGGGACAAAGG	Seed et al.[46]
	ICP3gp5F	ACCAACTCGACGCATAGCTT	
16S rDNA[1]	Maeda_1048_1067_F	GTGSTGCAYGGYTGTCGTCA	Maeda et al.[47]
	Maeda_1175_1194_R	ACGTCRTCCMCACCTTCCTC	
16S rDNA[2]	27F_Miseq	AATGATACGGCGACCACCGAGATCTACAC*TATGGTAATT*ccAGMGTTYGATYMTGGCTCAG	Chung et al.[28]
	338rcbc1	CAAGCAGAAGACGGCATACGAGAT**ACGAGACTGATT***AGTCAGTCAG*aaGCTGCCTCCCGTAGGAGT	

[1] 16S rDNA primer pair used for nanoliter qPCR. Degenerate primers are coded per standard convention (http://arep.med.harvard.edu/labgc/adnan/projects/Utilities/revcomp.html).
[2] Example of 16S rDNA primer pair used for microbiome analysis. Degenerate primers are coded per standard convention. Structure of forward primer: (i) 5' Illumina adapter, (ii) *Forward primer pad*, (iii) Forward primer linker (lower case), (iv) Forward primer. Structure of reverse primer example: (i) Reverse complement of 3′ Illumina adapter (underlined), (ii) Golay barcode (bold text), (iii) Reverse primer pad (italics), (iv) Reverse primer linker (lower case), (v) Reverse primer.

Table S2.

Table S2. Antibiotic impact on microbiota

	Any Antibiotic					Metronidazole					Ciprofloxacin					Azithromycin				
	Pos	Neg	Fold[1]	p[2]	p[3]	Pos	Neg	Fold[1]	p[2]	p[3]	Pos	Neg	Fold[1]	p[2]	p[3]	Pos	Neg	Fold[1]	p[2]	p[3]
PERMANOVA (Bray Curtis): N	34	9	---	0.057	---	29	29	---	**0.001**	---	16	24[4]	---	0.158	---	17	43	---	**0.041**	---
Absolute Abundance: CT																				
Vibrio cholerae	23.99	19.37	**-24.6**	**0.039**	---	22.90	22.08	-1.8	0.330	---	25.53	21.78	**-13.5**	0.074	---	23.40	21.78	-3.1	**0.042**	---
Total Bacteria (16S)	14.41	12.87	-2.9	0.098	---	14.03	13.43	-1.5	0.415	---	15.09	12.87	-4.7	**0.009**	---	14.00	13.79	-1.2	0.943	---
Relative Abundance: %[5]																				
Vibrio	3.00	12.00	-4.0	0.571	0.634	14.00	6.00	+2.3	0.133	0.148	3.50	4.00	1.1	0.571	0.961	0.68	17.00	**25.0**	**0.004**	**0.040**
Enterobacteriaceae[6]	2.00	2.00	1.0	0.654	0.654	6.00	0.82	**+7.3**	**0.017**	**0.038**	0.76	2.00	2.6	0.908	0.990	2.00	2.00	1.0	0.432	0.617
Streptococcus (early)	5.00	0.76	**+6.6**	**0.050**	0.100	6.00	2.00	+3.0	**0.033**	0.055	2.50	2.00	1.3	0.769	0.961	1.00	3.00	3.0	0.919	0.919
Enterococcus (early)	0.02	<0.00	+3.8	0.240	0.322	0.03	0.01	+4.6	0.085	0.106	0.02	0.01	2.4	0.990	0.990	0.01	0.02	2.3	0.730	0.811
Bacteroides (middle)	0.09	2.00	**-21.3**	**0.019**	0.095	0.05	0.46	**-9.5**	**0.011**	**0.037**	0.39	0.57	1.4	0.450	0.961	0.06	0.19	3.3	0.204	0.408
Prevotella (late)	0.37	22.00	**-59.5**	**0.030**	0.100	0.09	2.00	**-22.8**	**<0.001**	**<0.001**	2.55	7.00	2.7	0.448	0.961	0.48	0.69	1.4	0.682	0.811
Roseburia (late)	0.01	0.07	-6.2	0.155	0.259	0.01	0.05	**-7.8**	**0.0190**	**0.038**	0.11	0.07	1.4	0.63	0.961	0.01	0.04	3.2	0.164	0.408
Brachyspira	0.07	0.01	**+7.1**	**0.040**	0.100	0.03	0.02	+1.1	0.868	0.868	0.25	0.02	**13.3**	**0.024**	0.240	0.05	0.01	4.0	0.080	0.400
Blautia	0.04	0.19	-4.3	0.257	0.322	0.03	0.11	-3.3	**0.0458**	0.065	0.10	0.11	1.1	0.69	0.961	0.03	0.10	3.4	0.304	0.507
Ruminococcus	0.18	3.00	**-17.1**	**0.012**	0.095	0.16	1.00	**-6.3**	**0.0008**	**0.004**	0.65	1.50	2.3	0.229	0.961	0.01	0.04	3.8	0.150	0.408

[1] Fold change between antibiotic and no-antibiotic conditions. Fold changes greater than 5 fold are bold.
[2] Mann-Whitney U test. Significance assigned at $p < 0.050$ and designated by bold text.
[3] Adjusted p values to account for increased false discovery rate given multiple comparisons (N=10) using the Benjamini-Hochberg method [48].
[4] Sample size = 23 for qPCR analysis.
[5] Median percent value given. 'Early', 'middle' and 'late' are categories of genera seen during phases of recovery from cholera as previously described[16]. The ranking by mean relative abundance independent of stratification for each of the ten genera from top to bottom are 1, 2, 3, 7, 17, 5, 37, 6, 24, and 34, respectively.
[6] 16S analysis did identify Escherichia apart from other genera within the Enterobacteriaceae family. Enterobacteriaceae is grouped with 'early' based published data on Escherichia[16].

Text S1.

Supplementary Methods.

Microbiota analysis parameters. Qiagen CLC Workbench V13.8 parameters were as follows: (i) Minimum number of reads per sample = 100 and minimum percent from the median = 50.0. (ii) Quality limit = 0.05, ambiguous limit = 2, short reads were discarded, and minimum number of nucleotides in reads = 15. (iii) Reference based OTU clustering used the Greengenes Version 13.8 database, the threshold for taxonomic similarity = 97%, minimum occurrence = 2, chimera crossover cost = 3, Kmer size = 6, mismatch cost = 1, minimum score = 40, gap cost = 4, and maximum unaligned end mismatches = 5.
